# Elective Open Suprarenal Aneurysm Repair in England from 2000 to 2010 an Observational Study of Hospital Episode Statistics

**DOI:** 10.1371/journal.pone.0064163

**Published:** 2013-05-23

**Authors:** Alan Karthikesalingam, Peter J. E. Holt, Benjamin O. Patterson, Alberto Vidal-Diez, Giuseppe Sollazzo, Jan D. Poloniecki, Robert J. Hinchliffe, Matthew M. Thompson

**Affiliations:** 1 St George’s Vascular Institute, St. George’s University of London, London, United Kingdom; 2 Department of Community Health Sciences, St. George’s University of London, London, United Kingdom; Wayne State University School of Medicine, United States of America

## Abstract

**Background:**

Open surgery is widely used as a benchmark for the results of fenestrated endovascular repair of complex abdominal aortic aneurysms (AAA). However, the existing evidence stems from single-centre experiences, and may not be reproducible in wider practice. National outcomes provide valuable information regarding the safety of suprarenal aneurysm repair.

**Methods:**

Demographic and clinical data were extracted from English Hospital Episodes Statistics for patients undergoing elective suprarenal aneurysm repair from 1 April 2000 to 31 March 2010. Thirty-day mortality and five-year survival were analysed by logistic regression and Cox proportional hazards modeling.

**Results:**

793 patients underwent surgery with 14% overall 30-day mortality, which did not improve over the study period. Independent predictors of 30-day mortality included age, renal disease and previous myocardial infarction. 5-year survival was independently reduced by age, renal disease, liver disease, chronic pulmonary disease, and known metastatic solid tumour. There was significant regional variation in both 30-day mortality and 5-year survival after risk-adjustment. Regional differences in outcome were eliminated in a sensitivity analysis for perioperative outcome, conducted by restricting analysis to survivors of the first 30 days after surgery.

**Conclusions:**

Elective suprarenal aneurysm repair was associated with considerable mortality and significant regional variation across England. These data provide a benchmark to assess the efficacy of complex endovascular repair of supra-renal aneurysms, though cautious interpretation is required due to the lack of information regarding aneurysm morphology. More detailed study is required, ideally through the mandatory submission of data to a national registry of suprarenal aneurysm repair.

## Introduction

Since its development in 1991 [1], endovascular aneurysm repair (EVAR) has supplanted open repair (OR) as the most common treatment for infrarenal abdominal aortic aneurysms (AAA) in the UK [2] and USA [3]. This trend has been supported by randomised controlled trials, which demonstrated that EVAR was associated with considerably lower perioperative mortality than OR and equivalence in long-term survival [4]–[7]. The development of fenestrated (FEVAR) and branched devices has extended the endovascular approach to patients with more complex aneurysm morphology, with disease involving the renal artery origins (suprarenal aneurysms) or coeliac and superior mesenteric origins (Crawford type IV thoraco-abdominal aneurysms). However, the technique is relatively novel and technically challenging, while there remains uncertainty regarding the optimum use of FEVAR in juxtarenal (JRA), suprarenal (SRA) or type IV thoraco-abdominal (TAA) aneurysms [8]. Open surgery provides an accepted standard of care for these patients, and it has been suggested that the perioperative outcomes of open surgery should therefore be used as a benchmark for FEVAR [9].

The technique of FEVAR can be applied to JRA, SRA, and type IV TAA [10]. Outcomes of fenestrated aortic procedures should therefore be compared to those following open repair of all these types of aneurysm. Individual centres have reported that open JRA repair can be performed with perioperative mortality as low as 2.5–2.9% [9], [11], while data from the American National Surgical Quality Improvement Programme (NSQIP) suggested that complex aneurysms, defined as those with visceral involvement, can be repaired with similar mortality to infrarenal AAA (5.7% vs. 5.1%, respectively) [12]. Others have noted that SRA or type IV TAA repair was associated with significantly greater perioperative mortality than JRA repair (20% vs. 4.5%) [13], [14]. The existing data for open complex aneurysm repair stem largely from individual centres of excellence and are limited by small sample sizes, the potential for publication bias, and a lack of external validity. In contrast, administrative data provide an unselected and representative record of national outcomes. An epidemiological study of open suprarenal aneurysm repair in England is of particular value as a counterpart for the results of the GLOBALSTAR registry, which reported every FEVAR performed in the UK between 2007 and 2010 [15] (including 3-vessel or 4-vessel FEVAR SRA or type IV TAA). The present study aimed to place these data in context, by reporting and analysing the outcomes of open SRA repair during the same time period, in the same healthcare system.

## Methods

Demographic and in-hospital outcome data were extracted from Hospital Episode Statistics for patients undergoing elective open suprarenal aneurysm repair in England between 1 April 2000 and 31 March 2010. These data were linked to mortality records from the Office of National Statistics (ONS) to track long-term survival. The admission on which the procedure was performed was termed the index admission for the purposes of this study, and defined reporting standards were followed [16]. The HES are the administrative data set for the English National Health Service (NHS), which contains information regarding every admission of a patient to an NHS hospital. HES data are pseudonymized by the allocation of a unique identifier to each patient, so individuals can be tracked as their care moves from consultant to consultant (episodes) on any particular admission and between hospital admissions (spells). The data set therefore allows individual patients to be followed with respect to multiple hospital admissions. This feature is unique to the HES data over administrative data sets from other countries, which do not have a facility for long-term follow-up. The Office for National Statistics (ONS) is an independent, executive office of the UK Statistics Authority, which records the date of all deaths in the UK matched to individual demographic data. This includes deaths both in and out of hospital. The date of death of each patient recorded in the ONS registry is linked to the patient’s individual HES identifier. This allowed long-term outcomes to be quantified for the present cohort in terms of in- and out-of-hospital deaths. Each patient was matched against ONS records to identify any recorded date of death for 10 years following the index admission [17]. Owing to the uncertainty regarding aortic-related deaths, as few patients undergo autopsy in the UK, only all-cause mortality was reported in this study.

Elective suprarenal aneurysm (SRA) repairs were identified from the HES using in-house software as previously described [2], [18], [19]. The inclusion criterion was defined as all patients with an OPCS (Office of Procedures, Census and Surveys) procedural code of L19.3 (“Replacement of aneurysmal segment of suprarenal abdominal aorta by anastomosis of aorta to aorta”). A minimum age of 50 years was used to exclude miscoded paediatric cases with congenital aortic disease. Emergency admissions, urgent admissions and patients with thoracic, infrarenal or ruptured aneurysms, were excluded. Patients known to have undergone bypass, ligation, endarterectomy, embolization, or “other operation on” visceral branches of the abdominal aorta were excluded; in an attempt to restrict the study inclusion criterion to true suprarenal aneurysms without visceral segment involvement. However, a limitation of this study was the lack of data or imaging regarding aneurysm morphology, and the incorporation of some patients with infrarenal AAA or type IV thoraco-abdominal aneurysms could not be definitively guaranteed.

The primary outcome measure was in- and out-of-hospital all-cause mortality at 30 days. The secondary outcome measures were length of stay, and 5-year all-cause survival, with a sensitivity analysis for the effect of the perioperative period on long-term outcome performed by including and excluding those who survived the first 30 days.

Statistical models were constructed to identify the relative importance of demographic, geographic and comorbidity indicators on these outcomes (both 30-day mortality and 5-year survival). The modelling included data regarding each patient’s year of surgery, the Strategic Health Authority in which the index procedure was performed (SHA; ten geographical regions of healthcare provision in the English National Heath Service) patient demographics (age, gender and social deprivation index) and comorbidity. An interaction term for SHA and social deprivation was included in all statistical models, as there are well-established geographical variations in social deprivation in the UK, which tend to overlap with areas of poor healthcare outcomes [20]. Co-morbidity was identified using individual components of the Royal College of Surgeons Charlson score methodology for administrative data [21], and categorised as prior myocardial infarction, congestive cardiac failure, peripheral vascular disease, cerebrovascular disease, chronic pulmonary disease, diabetes mellitus, renal disease, dementia or previous malignancy. The annual caseload (volume) of SRA repairs undertaken in each hospital per year were incorporated in all models by grouping hospitals into quintiles of caseload. The quintiles comprised five groups of institutions, arranged by the number of SRA repairs undertaken per year, each quintile containing a similar number of patients. They were created to ensure that all hospitals of a particular annual operative caseload were grouped together, rather than splitting hospitals of the same annual volume to create exactly the same number of patients in each quintile.

### Statistical Analysis

All analyses were performed using SAS version 9.1 (SAS Institute Inc., USA).

#### 1. 30-day mortality

Multivariate analysis was performed using a binary logistic regression model. A backward selection procedure was used, incorporating variables significant in univariate analysis at p<0.1. Inclusion in the model required a significance level of α = 0.1. Significant results were reported from the model at a significance level of α = 0.05.

#### 2. 5-year survival (including and excluding 30-day mortality)

Kaplan-Meier analysis was used to report all-cause survival. Subgroup comparison was performed by log-rank test, with Sidak adjustment for multiple comparisons. Cox proportional hazards models were fitted for analysis of survival, including consideration of gender, age on index admission, quintiles of social deprivation, strategic health authority (SHA), year of surgery, and comorbidity. Backward and stepwise selection procedures were used, with comparison of models by Akaike’s Information Criterion (AIC) to ascertain whether individual covariates improved goodness-of-fit. Inclusion in the model required a significance level of α = 0.1, and significant results were reported at α = 0.05.

#### 3. Length of stay

For length of stay, multiple linear regression was performed on the logarithm of the length of stay, using a general linear model procedure. Type III (orthogonal) sum of squares analyses were tested using the F-distribution, and the effect of independent variables on length of stay was quantified using regression estimates.

#### 4. Comparison between different strategic health authorities or between quintiles of social deprivation index

Comparison of risk-adjusted outcomes was performed by planned orthogonal contrasts between the ten SHAs, or between the five quintiles of social deprivation index.

## Results

**Figure 1:**
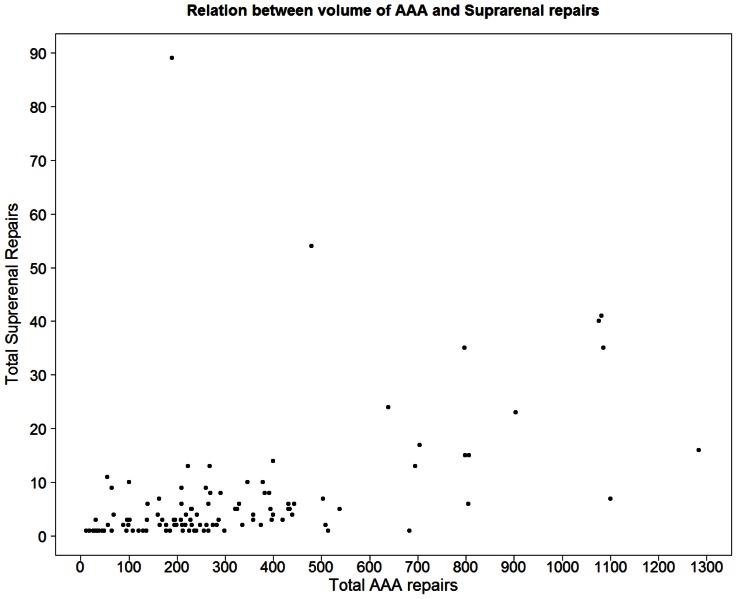
Provision of suprarenal aneurysm repair in English hospitals from 2000–2010, compared to provision of infrarenal repair during the same period.

793 SRA repairs were performed in England between 1 April 2000 and 31 March 2010. 609/793 (76.8%) patients were male, with a mean ± s.d. age of 71.6±7.11 years. Mean ± s.d. follow-up was 3.52±2.90 years; and comorbidity was common with a history of myocardial infarction in 99/793 (12.48%) cases, congestive cardiac failure in 70/793 (8.83%), chronic obstructive pulmonary disease in 147/793 (18.54%) and renal disease in 120/793 (15.13%) (Table 1). SRA repair was distributed unevenly amongst UK trusts (Figure 1). 80% of all hospitals performing SRA repair undertook less than one SRA repair per year over the study period and 223/793 cases (28%) were recorded at 4 hospitals in Newcastle, London (n = 2) and Bristol (Figure 1).

**Table 1:** Characteristics of patients undergoing suprarenal aneurysm repair in England from 2000–2010.

Patient characteristic	Mean (SD) or n(%)
Age	71.61 (7.11)
Gender	612 (77.18%) Male181 (22.82%) Female
Comorbidity	
* Myocardial infarction*	99 (12.48%)
* Congestive cardiac failure*	70 (8.83%)
* Peripheral vascular disease*	764 (96.34%)
* Cerebrovascular disease*	66 (8.32%)
* Dementia*	6 (0.76%)
* Chronic pulmonary disease*	147 (18.54%)
* Rheumatological disease*	18 (2.27%)
* Liver disease*	10 (1.26%)
* Diabetes mellitus*	71 (8.95%)
* Hemiplegia or paraplegia*	11 (1.39%)
* Renal disease*	120 (15.13%)
* Any malignancy*	94 (11.85%)
* Metastatic solid tumour*	12 (1.51%)

### Thirty-day Mortality

**Figure 2:**
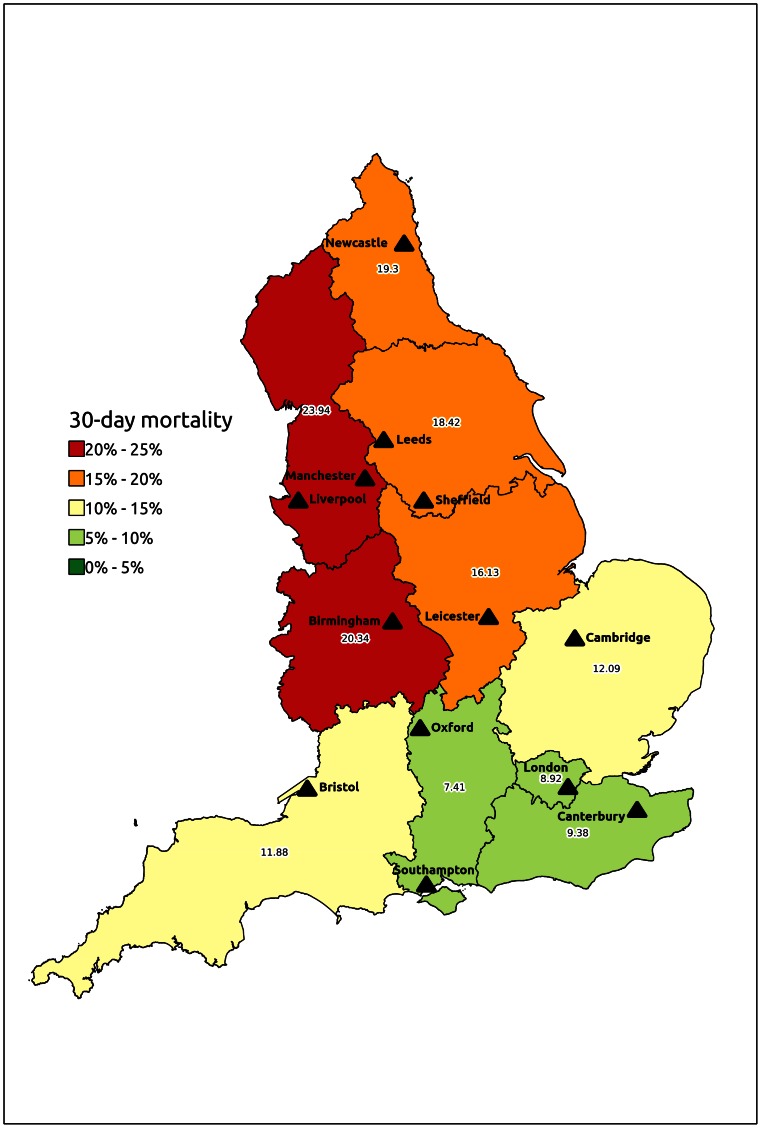
Map to illustrate 30-day mortality after elective suprarenal aneurysm repair in different Strategic Health Authorities in England between 2000–2010. Indicated cities contain the hospital performing most aneurysm repairs in each Strategic Health Authority.

**Figure 3:**
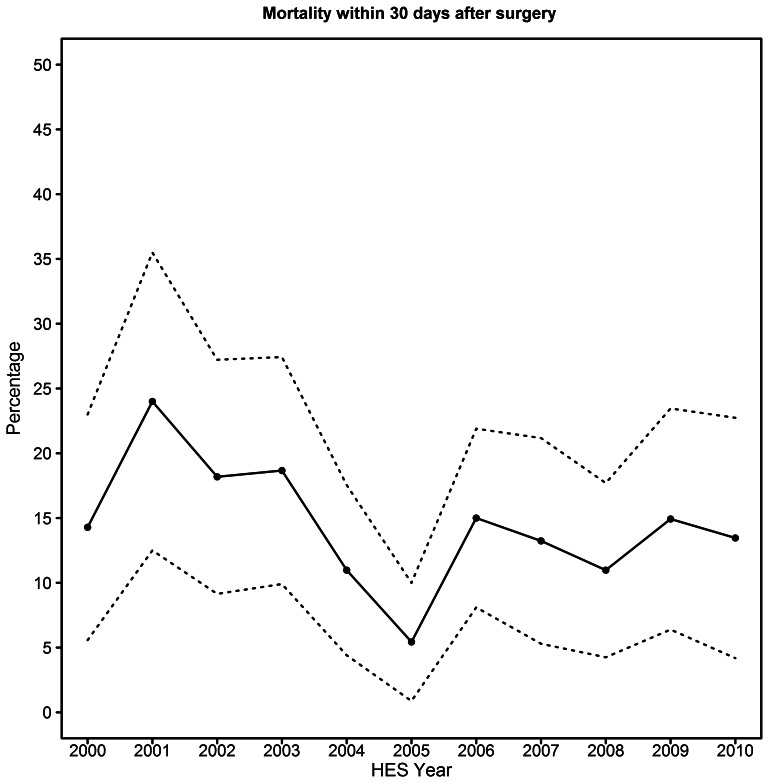
30-day mortality after suprarenal aneurysm repair in England from 2000 to 2010, with 95% confidence intervals.

Thirty-day mortality was 111/793 patients (14.0%). Age (OR 1.050, 95% CI 1.018–1.082, p = 0.0021), previous myocardial infarction (OR 1.837, 95% CI 1.021–3.306, p = 0.0424), pre-existing renal disease (OR 2.194, 95% CI 1.348–3.650, p = 0.0017) and SHA (p = 0.0191) were independent predictors of thirty-day mortality in logistic regression modelling. An interaction term for SHA*social deprivation quintile was not significant, and social deprivation was not a significant independent predictor of thirty-day mortality. Two subgroups of SHAs were identified through orthogonal contrasts analysis, with no significant difference between the 30-day mortality of SHAs contained within one subgroup. A significantly higher death rate was associated with surgery in the subgroup containing North East, North West, Yorkshire and the Humber and West Midlands SHAs, compared to the subgroup containing East Midlands, East of England, London, South East, South Central and South West SHAs (OR = 2.194, 95% CI 1.369–3.515) (Figure 2). There was no significant difference in 30-day mortality between each year of surgery from 2000 to 2010 (Figure 3), or between quintiles of hospital caseload (crude mortality rates were 10.3%, 9.1%, 16.7%, 22.7% and 15.5% in quintiles 1 to 5 respectively). A sensitivity analysis for mortality in the largest 4 hospitals (according to surgical caseload) also demonstrated no difference in 30-day mortality (23/223 vs 88/570, p = 0.07).

### Five-year Survival

**Figure 4:**
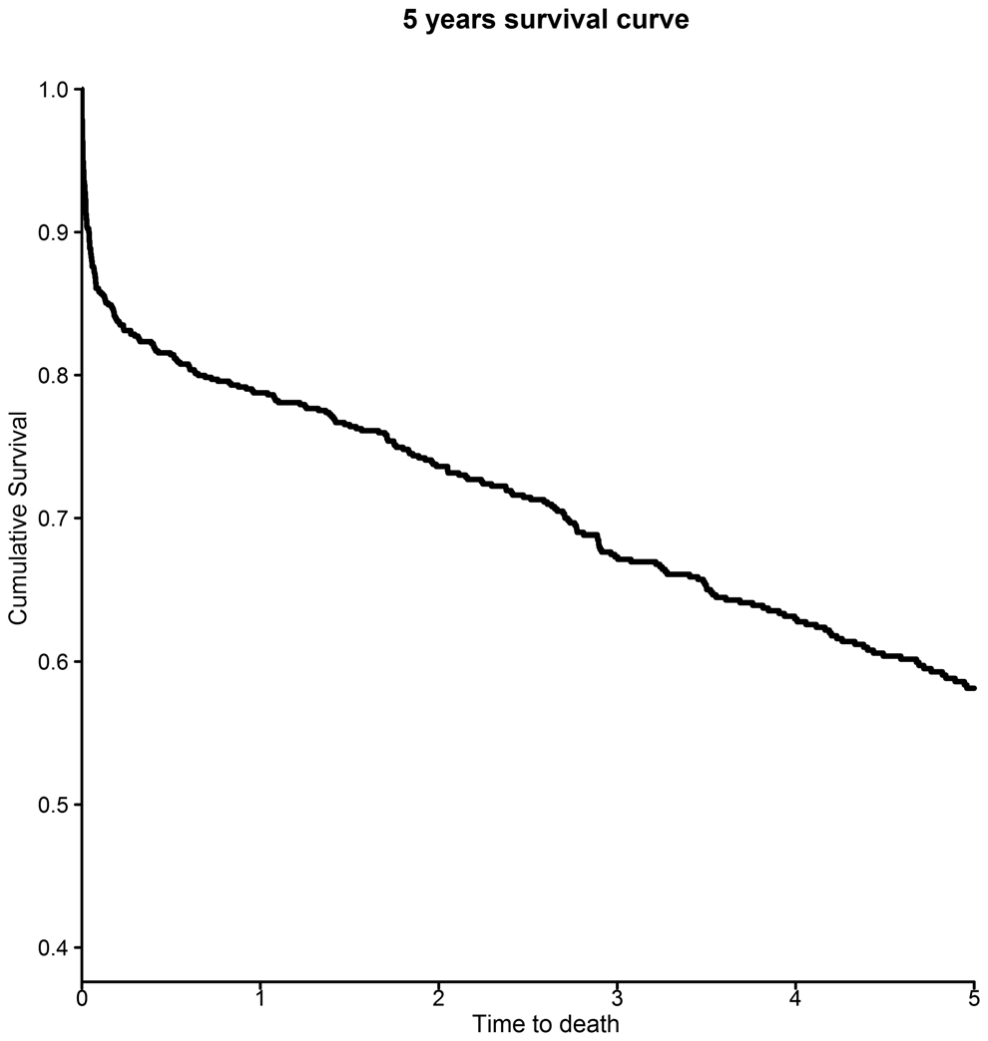
Kaplan-Meier plot of all-cause survival after suprarenal aneurysm repair in England from 2000 to 2010.

**Figure 5:**
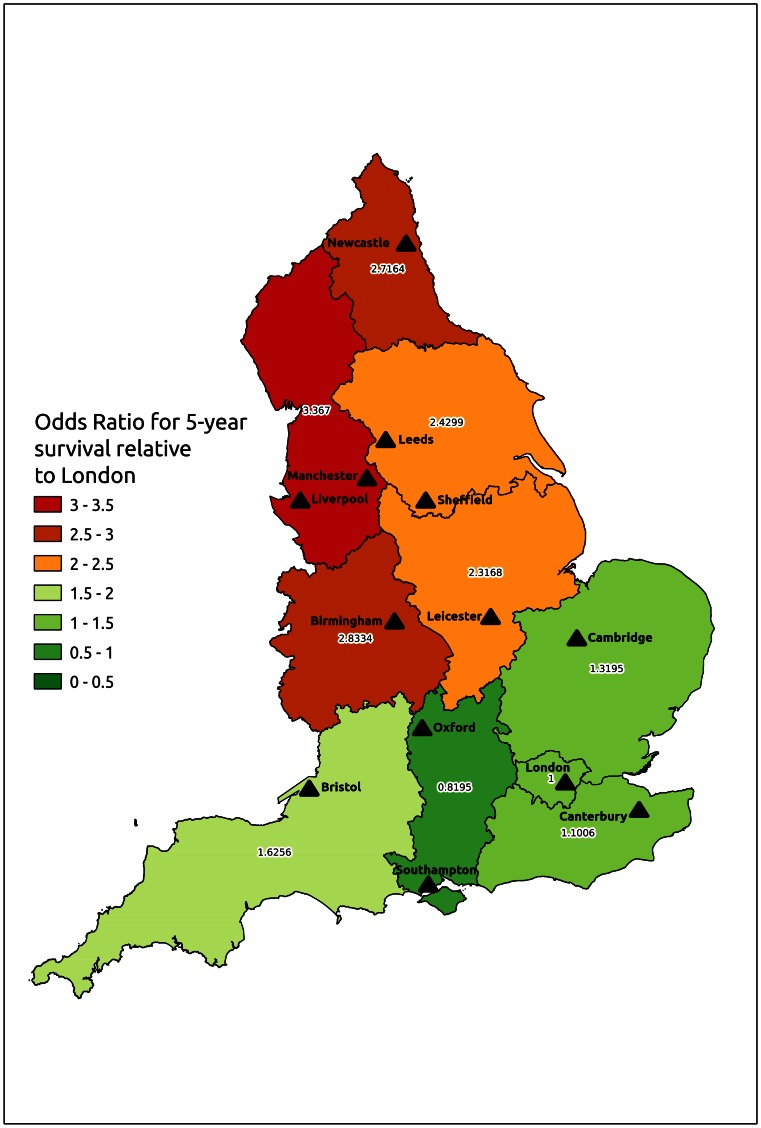
Map to illustrate the odds ratio for 5-year risk-adjusted survival after elective suprarenal aneurysm repair in different Strategic Health Authorities in England between 2000–2010, in relation to 5-year survival in London. Indicated cities contain the hospital performing most aneurysm repairs in each Strategic Health Authority.

Freedom from all-cause mortality was 78%, 73%, 67%, 63% and 58% in years 1, 2, 3, 4 and 5 after surgery (Figure 4). The Cox Proportional Hazards model for 5-year survival from the day of surgery demonstrated that age (HR 1.03, 95% CI 1.01–1.05, p = 0.0005), chronic obstructive pulmonary disease (HR 1.42, 95% CI 1.07–1.88, p = 0.016), liver disease (HR 2.46, 95% CI 1.09–5.59, p = 0.0310), renal disease (HR 1.80, 95% CI 1.34–2.41, p<0.0001), metastatic solid tumour (HR 3.06, 95% CI 1.43–6.49, p = 0.0038) and Strategic Health Authority (p = 0.0138) were predictive of poor survival, with poorer 5-year survival in the North East, North West, Yorkshire and West Midlands regions than in the South East, South West or South Central regions (Table 2) (Figure 5). An interaction term for SHA*social deprivation quintile was not significant, and neither social deprivation nor annual hospital SRA volume was a significant independent predictor of five-year survival.

**Table 2:** Significant covariates in Cox Proportional Hazards Model for 5-year survival from the day of elective open suprarenal aneurysm repair.

Parameter	Hazard Ratio	95% Confidence Limits		p-value
**Age on admission**	1.03	1.01	1.05	0.0005
**Chronic pulmonary disease**	1.42	1.07	1.88	0.0160
**Liver disease**	2.46	1.09	5.59	0.0310
**Renal disease**	1.80	1.34	2.41	<0.0001
**Metastatic solid tumour**	3.06	1.43	6.49	0.0038
**Strategic Health Authority**				
* North East vs South East*	2.274	1.034	4.999	0.0138
* North East vs South Central*	2.499	1.239	5.041	0.0138
* North East vs South West*	1.743	1.037	2.929	0.0138
* North West vs South East*	2.346	1.073	5.129	0.0138
* North West vs South Central*	2.579	1.280	5.196	0.0138
* North West vs South West*	1.798	1.072	3.017	0.0138
* Yorkshire and the Humber vs South East*	2.266	1.043	4.924	0.0138
* Yorkshire and the Humber vs South Central*	2.491	1.251	4.959	0.0138
* Yorkshire and the Humber vs South West*	1.737	1.056	2.857	0.0138
* West Midlands vs South East*	2.478	1.130	5.435	0.0138
* West Midlands vs South Central*	2.724	1.353	5.481	0.0138
* West Midlands vs South West*	1.900	1.132	3.188	0.0138

### Five-year Survival in those Remaining Alive after the First 30 Postoperative Days

**Figure 6:**
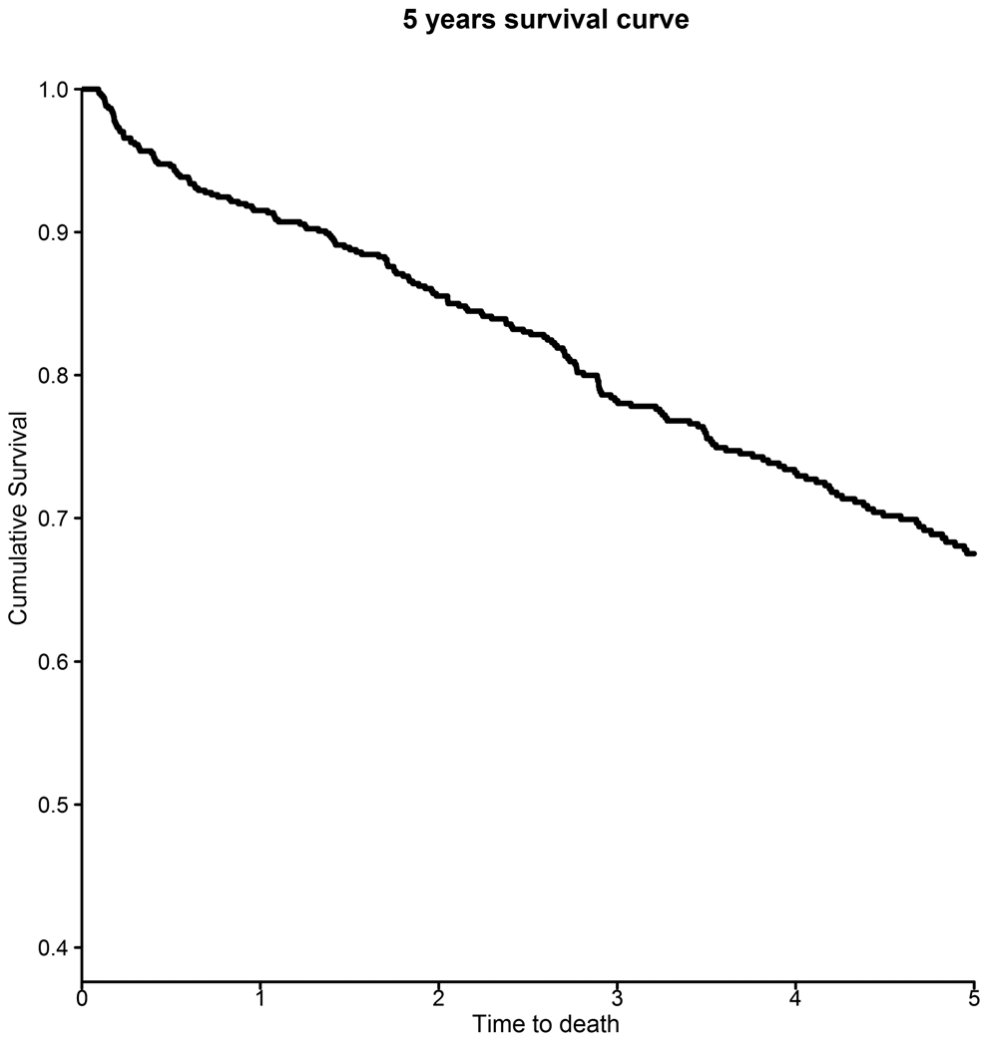
Kaplan-Meier plot of all-cause mortality in survivors of the first 30 days after suprarenal aneurysm repair.

In patients surviving the first 30 postoperative days, freedom from all-cause mortality was 91%, 86%, 78%, 73% and 68% in years 1, 2, 3, 4 and 5 (Figure 6). The Cox Proportional Hazards model for 5-year survival in this group demonstrated that age (HR 1.04, 95% CI 1.01–1.06, p = 0.0041), dementia (H.R. 8.55, 95% CI 2.65–27.78, p = 0.0003), chronic obstructive pulmonary disease (HR 1.93, 95% CI 1.37–2.73, p = 0.0002) liver disease (HR 3.83, 95% CI 1.18–12.50, p = 0.0253), renal disease (HR 1.63, 95% CI 1.10–2.42, p = 0.0158), metastatic solid tumour (HR 5.10 95% CI 2.28–11.36, p<0.0001) and social deprivation index (p = 0.0052) were predictive of poor survival, with poorer 5-year survival in the lowest two quintiles of social deprivation index (Table 3). There was no significant interaction between social deprivation index and SHA. SHA was not a significant independent predictor of 5-year survival in those remaining alive after the first 30 postoperative days.

**Table 3:** Significant covariates in Cox Proportional Hazards Model for 5-year survival in survivors of the first 30 days after elective open suprarenal aneurysm repair.

Parameter	Hazard Ratio	95% Confidence Limits		p-value
**Age on admission**	1.035	1.011	1.059	0.0041
**Dementia**	8.55	2.65	27.78	0.0003
**Chronic pulmonary disease**	1.93	1.37	2.73	0.0002
**Liver disease**	3.83	1.18	12.50	0.0253
**Renal disease**	1.63	1.10	2.42	0.0158
**Metastatic solid tumour**	5.10	2.28	11.36	<.0001
**Social Deprivation Index Quintiles**				
* 1 vs 3*	1.862	1.135	3.054	0.0052
* 1 vs 5*	2.278	1.381	3.756	0.0052
* 2 vs 3*	1.652	1.011	2.698	0.0052
* 2 vs 5*	2.021	1.232	3.315	0.0052

### Length of Stay

Patients with peripheral vascular disease (p<0.0001) and cerebrovascular disease (p = 0.0350) had a longer length of stay than those without. SHA was also a significant predictor of length of stay(p = 0.0350), presumably as a result of the demonstrated difference in 30-day mortality.

## Discussion

Open SRA repair in England was performed with considerably greater mortality than has been reported for FEVAR in suprarenal and complex aneurysms [15], with evidence of considerable regional variation. Although it has been suggested that SRA repair (with or without visceral revascularisation) can be performed with equivalent safety to infrarenal AAA repair [12], the thirty-day mortality of SRA repair in this study was considerably higher than that reported for infrarenal AAA [2], [18] or open JRA repair (2.9%) [9] and was similar to the rate that has been reported by many groups for the open repair of SRA and type IV TAA (5% to 22%)[13], [22]–[27]. Detailed information regarding aneurysm morphology was not available in the present study, and it is not possible to definitively exclude the analysis of some cases involving the visceral aorta in these results. The present study adds weight to the argument that more proximal aneurysmal disease is associated with greater perioperative mortality after open repair [13], with the requirement for supravisceral clamping implicated in greater morbidity and mortality than a suprarenal aortic clamping [14]. After risk adjustment for comorbidity and demographics, the rate of perioperative mortality did not improve over the study period (Figure 3), despite assertions that developments in complex aneurysm repair have increased its safety in recent years [26] alongside evidence that increasingly elderly patients are being treated for AAA in England [28].

It has been suggested that the results of open JRA repair should be used to provide context for the performance of FEVAR [11], but FEVAR can also be employed to treat the suprarenal and visceral aorta. The present study therefore adds useful evidence to the open surgical benchmark against which FEVAR must be judged: it is important that the results of open surgery for SRA or type IV TAA should be considered alongside the data for open repair of JRA, to acknowledge the full range of disease that can be treated by FEVAR. Patients undergoing open SRA repair, as presented in this study, provide a useful comparative group for patients who have undergone 3-vessel or 4-vessel FEVR, with sealing zones above the superior mesenteric or coeliac artery. Previous evidence describing open repair of SRA and type IV TAA has been limited by small sample sizes, the inclusion of symptomatic or ruptured cases alongside elective repairs, and mixed cohorts of patients with JRA and SRA (despite differences in the outcomes of surgery for JRA, SRA or type IV TAA) [26].

Administrative data, as provided by the present study, are of particular value in reflecting surgical outcomes because single-centre results are open to publication bias and can outperform wider national practice [27]. The present study is of particular value as a counterpart to the results of the BSET (British Society of Endovascular Therapy) FEVAR registry, which reported that 318 FEVARs were performed in the UK between 2007 and 2010 with 4.1% perioperative mortality. The BSET investigators reported that increasing the number of fenestrations for FEVAR led to greater mortality, although this observation did not reach statistical significance (a finding most likely explained by type II error). Mortality was 2.7% after FEVAR with renal fenestrations alone, and 2.9% for grafts with a superior mesenteric scallop/fenestration. Mortality was 9.4% for FEVAR incorporating the coeliac trunk; this subgroup is morphologically similar to aneurysms treated by open SRA repair and the result can be directly compared to the present study, where mortality was 14%. Overall, the BSET results suggest that FEVAR compares favourably with open SRA repair.

In the present study, increasing age, renal disease and previous myocardial infarction were independent predictors of 30-day mortality, which was consistent with existing evidence. Age and renal function have previously been shown to exert an important effect on perioperative outcome for SRA [12], [13] and estimated glomerular filtration rate (eGFR) in particular has been shown to effect outcome in many atherosclerotic cardiovascular conditions [29]–[31]. Renal function is important in SRA repair as the requirement for suprarenal aortic clamping during the operation imposes a period of renal hypoperfusion, which is less tolerated by patients with pre-existing renal impairment. In this study “Renal Disease” was defined according to the RCS Charlson index; a validated tool that defines renal impairment (and other comorbidity) according to pre-specified administrative diagnostic codes. Biochemical data such as eGFR would have provided a more precise indicator of renal function but were not available in HES.

Predictors of long-term mortality included age, chronic pulmonary disease, renal disease, liver disease and metastatic solid tumour; after removal of the perioperative deaths, social deprivation index replaced strategic health authority as a predictor of long-term survival. These indicators represent potential areas for targeted improvement of the 5-year outcome for patients with repaired aneurysms, as despite recent evidence that aortic aneurysm prevalence has fallen with improvements in public health [28], [32], the fate of patients who survive SRA repair is concerning.

There was a significant difference in mortality across different geographical regions in England (Figure 2) after risk adjustment. Similar regional variation was seen in five-year survival (Figure 5) but was eliminated by restricting analysis to survivors of the first 30 postoperative days. This suggested that perioperative outcome was of key importance in determining the long-term survival of patients with SRA in different regions of England. The excellent results of complex open aneurysm repair in Scotland have been partly attributed to the concentration of national expertise in a single centre [26], and the extent of regional variation observed in the present study suggests that further data are required to inform debate regarding the configuration of complex aneurysm surgery in England, both in terms of regional variation and case volume.

**Figure 7:**
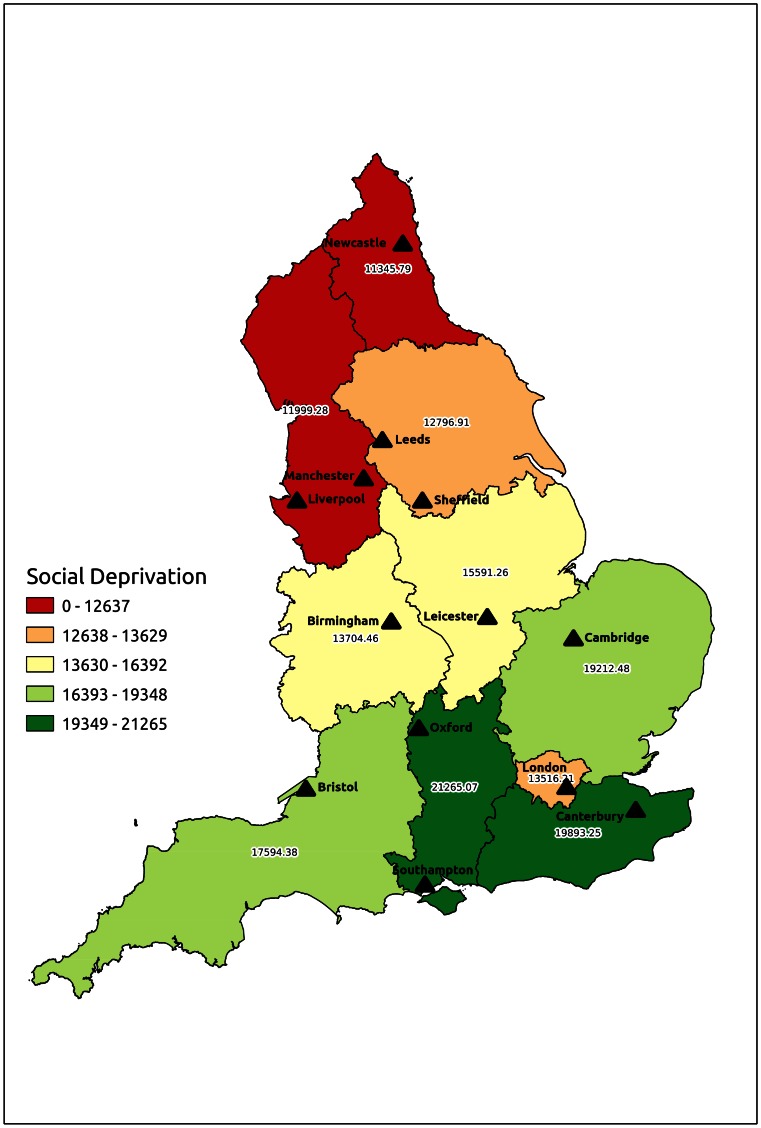
Map to illustrate mean social deprivation index of patients undergoing elective suprarenal aneurysm repair in different English Strategic Health Authorities.

Regional variation in risk-adjusted mortality has been demonstrated for a range of disease conditions and cardiovascular procedures in England [20]. The differing outcomes of SRA repair in different SHAs was striking, but should be interpreted cautiously given the limitations of this study. The worst outcomes were seen in the North and Northwest regions of England; raising the likelihood of incomplete risk adjustment for confounding differences in underlying health. Social deprivation and analysis by SHA may actually have been indices of a generally less healthy population in the North of England, rather than variation in the quality of SRA repair services. Inequalities in life expectancy between the North and South of England have been well-documented, with northern regions believed to fare poorly due to a complex blend of socioeconomic, environmental, educational, genetic and lifestyle factors [33]. Only one administrative index of social deprivation was used for this study, and many other factors may have been incompletely quantified, including smoking behaviour, physical activity and cardiovascular risk prevention therapies. A map of social deprivation in patients undergoing SRA repair highlights the underlying “North-South divide” between regions, with the anomaly that patients in London had greater social deprivation than other southern SHAs (Figure 7). However, after risk-adjustment there was no demonstrable statistical interaction between SHA and social deprivation in this study.

Case volume was not a significant predictor of outcome in this study, but there were a paucity of events for study in each volume quintile and type II error cannot be excluded. Alongside the inability to validate or adjust for differences in case mix, turndown rates and variability in coding; the limited sample available for study should prevent further inferences for or against further regionalisation of SRA care from the present data sample. The high overall mortality seen in this study is concerning, but might reflect the inclusion of type IV thoracoabdominal TAAAs alongside true SRAs. Confounding may also have been introduced by differences in regional coding, and the major limitations of this study relate to the nature of the Hospital Episode Statistics (HES) dataset. Operative detail was restricted by the use of OPCS-4 procedure codes in HES and it was not possible to obtain more precise data regarding the location of the aortic cross-clamp or aneurysm morphology from CT aortograms, to more precisely understand the types of aneurysm treated. OPCS-4 procedural codes used in the HES describe elective aneurysm repair for the infrarenal aorta (L19.4), suprarenal aorta (L19.3), thoracic aorta (L19.2) or ascending aorta (L19.1); there are no codes to indicate thoraco-abdominal aneurysms or classify these by Crawford subgroups. The present series is therefore likely to include both SRA and type IV TAA, which may explain the higher mortality observed in comparison to previous data combining JRA with SRA. Although this limits the usefulness of the present study as a specific reflection of SRA repair in distinction from type IV TAA repair, the combined data remain important as a benchmark against which 3-vessel and 4-vessel FEVAR can be judged.

The present study suggests that outcomes from open SRA repair in England may have scope for improvement, and are notably worse than national results for open infrarenal AAA repair. The data are supportive of the continuing development of FEVAR in specialist centres. In parallel, more detailed examination of open SRA repair is required in England, and a national registry incorporating detailed morphological and procedural data would allow further instructive comparison with the national FEVAR registry (GLOBALSTAR). The infrastructure for data collection already exists in the UK through the National Vascular Database [34], which could be readily modified for this purpose.

### Conclusion

This study of national outcomes across England demonstrates relatively high mortality after elective open suprarenal aneurysm repair, with significant regional variation in perioperative and longer-term survival. These data are useful as a benchmark for the comparison of national outcomes from 3-vessel or 4-vessel FEVAR. The outcomes of open suprarenal aneurysm repair in England require closer scrutiny, ideally through the mandatory submission of all referrals (both operated and unoperated) to a national registry reporting details of patient characteristics, operative technique, and aneurysm complexity.
